# Pig Manure Application for Remediation of Mine Soils in Murcia Province, SE Spain

**DOI:** 10.1100/tsw.2008.98

**Published:** 2008-08-30

**Authors:** A. Faz, D. M. Carmona, A. Zanuzzi, A. R. Mermut

**Affiliations:** ^1^ Sustainable Use, Management and Reclamation of Soil and Water Research Group, Agrarian Science and Technology Department, Technical University of Cartagena, Paseo Alfonso XIII, 52, 30203 Cartagena, Murcia, Spain; ^2^ Department of Soil Science, University of Saskatchewan, 51 Campus Drive, Saskatoon, SK S7N 5A8, Canada

**Keywords:** acid drainage, column leaching experiment, mine soil, marble waste, pig manure, remediation, Spain

## Abstract

In southern Spain, specifically in Murcia Province, an increased pig population causes large amounts of slurry production that creates a very serious environmental concern. Our aim was to use this waste to reduce the acid mine drainage process, heavy metal mobilization, and to improve soil conditions to enhance plant establishment in mine soils. Pig manure, sewage sludge, and lime were used as soil amendments in a field experiment and in undisturbed soil column. Field experiments showed an increase in pH, total nitrogen, organic carbon, and carbonate contents; a reduction of diethylene-tetramine pentaacetic acid (DTPA)– and water-extractable metals; and an improvement of plant establishment. The field studies showed that pig manure could be utilized to remediate polluted soils. Column studies in the laboratory showed that amendment of mine soil with pig manure initially increased soil pH from 2.21 to 6.34, promoted reduced conditions in the surface soil, and decreased the metal mobility. After 21 weeks, while the leachate was slightly acidic, however, the mobility of metals was substantially low. Additions of 7 and 14% of pig manure were insufficient to maintain a neutral pH in the leachate. Therefore, continuous application of the pig manure may be advised.

## INTRODUCTION

For more than 2,500 years, until 1991, intense mining activities carried out in the Cartagena-La Unión area, in Murcia Province (SE Spain), have caused a high accumulation of heavy metals and the generation of acid mine drainage into the environment. One of the techniques used to mitigate acid mine drainage is the addition of alkaline materials, which serve to either neutralize the acids or stop the oxidation of pyrite[1,2,3,4].

Mine soils have high contents of toxic metals that prevent or restrict plant growth. These soils are also very deficient in nitrogen and phosphorous, and exhibit unfavorable physical properties[5]. Due to the absence of vegetation cover, these soils are very sensitive to erosion, and they are the major source of heavy metal pollution for urban and agricultural areas[6]. They may also have detrimental effects on crops and public health. Therefore, there is an urgent need to stabilize these lands.

A range of reclamation techniques are available for mine soils. Due to high transportation costs and availability, covering them with imported soil can be very expensive. Recent reports[e.g., 7] suggested that long-term rehabilitation of mine soils can be achieved through the establishment of vegetation. Although revegetation is desirable, these mine soils have unfavorable conditions for plant growth, such as the high amount of residual heavy metals, macronutrient deficiencies, high acidity, reduced water retention, and poor physical characteristics. In order to revegetate this area, the addition of organic matter was required to raise the pH and remediate nutrient deficiencies.

The aim of this research was to evaluate the effect of pig manure on heavy metal mobility and some soil characteristics in the mining area of Cartagena-La Unión, SE Spain. To reduce acid mine drainage and heavy metal mobilization, and to improve soil conditions for plant establishment, we amended the soil with pig manure, sewage sludge, and lime in the field studies, whereas only pig manure was used in column leaching experiments.

## MATERIALS

### Study Area

The study area included two representative mine ponds from the mining district of the Cartagena-La Unión (110-0 m asl; 37º37′20″N, 0º50′55″W; 37º40′03″N, 0º48′12″W), located on the east side of Murcia province, SE Spain (Fig. 1). The soil was classified as Haplic Torriarents[8], and had a high amount of iron oxides and compacted subsurface hardpan. These ponds posed risks associated with acidic and saline conditions, and a high concentration of heavy metals (Table 1)[9,10,11].

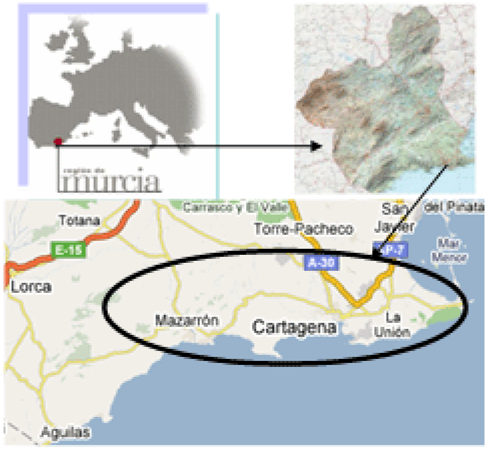

**FIGURE 1**. The location of the area studied.

### Amendments

Three types of waste materials were used as amendments: marble wastes or mud (lime), sewage sludge, and pig manure. Table 2 summarizes some characteristics of these materials. Lime was obtained from marble industries located in Cehegín (Murcia) and had approximately 0.5% humidity and 94% $CaCO_3$. Sewage sludge generated in the treatment plant of Cartagena had 80% humidity, 0.1% $CaCO_3$, and 8.8 g/kg total

**TABLE 1**
**Average pH, Electrical Conductivity (EC), and Total Nitrogen (TN), Organic Carbon (OC), and Total Extractable Metal Contents in the Soil from Plots and Undisturbed Soil Columns**

| | pH | EC | $EqCaCO_3$ | TN | OC | Total-extractable metals | | | |
|---|---|---|---|---|---|---|---|---|---|
| | | | | | | Zn | Pb | Cd | Cu |
| | | (dS $m^{-1}$) | (%) | (g $kg^{-1}$) | (g $kg^{-1}$) | (mg $kg^{-1}$) | | | |
| Soil from plots | 6.3 | 10.0 | 0.6 | 0.1 | 1.9 | 6763.7 | 2476.9 | 28.8 | 87.4 |
| Soil from column | 2.2 | 14.3 | 0.5 | 0.1 | 0.6 | 3403.9 | 9650.5 | 237.8 | 14.7 |

**TABLE 2**
**Chemical Properties of Pig Manure, Marble Mud, and Sewage Sludge**

| | | pig manure | marble mud | sewage sludge | | pig manure | marble mud | sewage sludge |
|---|---|---|---|---|---|---|---|---|
| pH $_{H2O}$ | | 7.79 | 8.39 | 7.73 | | Total metals, mg $kg^{-1}$ | | |
| EC $_{1:5}$ | dS $m^{-1}$ | 9.31 | 1.6 | 5.58 | Cd | 1.10 | 1.64 | 27.48 |
| Moisture | % | 13.5 | 0.5 | 79 | Cu | 1212 | 6.69 | 215.5 |
| Total nitrogen | g $kg^{-1}$ | 28.1 | 0.1 | 8.8 | Pb | 68.08 | 6.24 | 591 |
| Organic carbon | g $kg^{-1}$ | 34.0 | 0 | 33.6 | Zn | 972 | 2.74 | 3477 |
| $CaCO_3$ | % | 5.7 | 93.5 | 0.1 | | | | |
| Exchange capacity | cmol(+) $kg^{-1}$ | 38.8 | 2.5 | 67.5 | | | | |

EC: electric conductivity nitrogen. The pig manure came from a pig farm located in Fuente Álamo (Murcia), with a 13.5% humidity, 5.7% $CaCO_3$, and 28.1 g/kg total nitrogen.

## Field Experiment

Field plots (4 $m^2$ each) were established in selected mine soils (Figs. 2 and 3). Three doses of pig manure and sewage sludge were used to provide nutrients and organic matter to these soils; and only one dose of lime was added to raise the pH. Pig manure doses were 6.25 (P1), 12.5 (P2), and 25 t/ha (P3), whereas sewage sludge doses consisted of 5 (S1), 10 (P2), and 20 t/ha (S3).

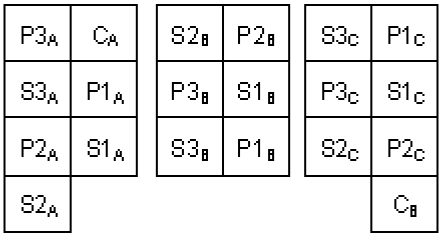

**FIGURE 2**. Layout of the plots. C: control; pig manure doses P1-P2-P3; sewage sludge doses S1-S2-S3. A, B, and C: replicates of each treatment.

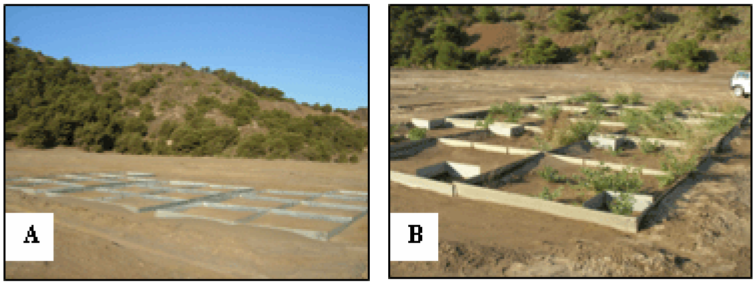

**FIGURE 3.** Experimental plots: (A) before amendments and (B) after 12 months of application.

Soil samples were taken from the surface and subsurface layers of experimental plots after 12 months of amendment addition. Soil pH was measured as 1:1 in water/soil ratio[12]. Equivalent calcium carbonate was determined using Bernard calcimeter. Total nitrogen was determined using the Kjeldahl method[13]. Organic carbon was analyzed using a Total Organic Carbon Analyser (TOC-V CSH Shimadzu). Water- and DTPA-extractable Zn, Pb, Cd, and Cu were determined using AAS (UNICAM 969) as suggested in the literature[14,15,16].

## Column Leaching

Undisturbed soil columns were taken from the areas affected by mining activities in the vicinity of the plots. The equipment shown in Fig. 4 was used to collect undisturbed soil columns. They were made of transparent metacrylate and were 60 cm long and 25 cm inside diameter. The undisturbed soil in each column was 35 cm long.

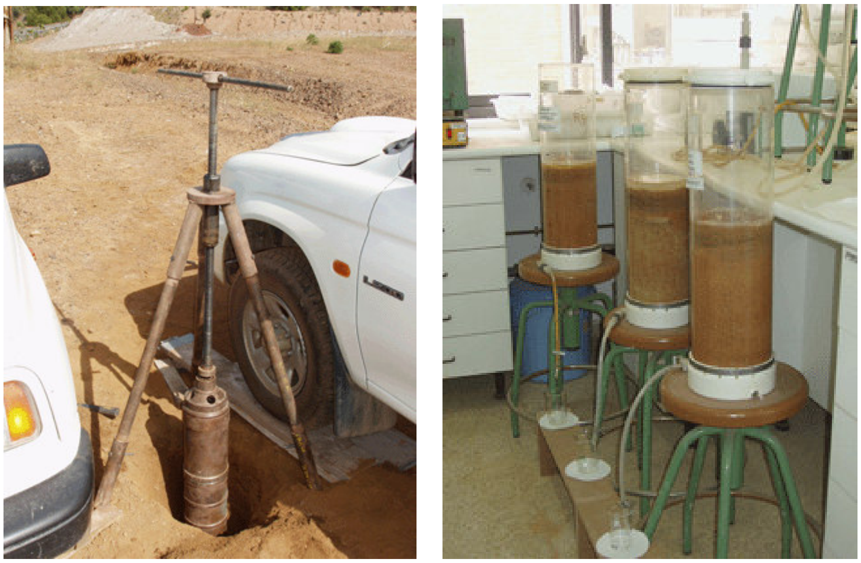

**FIGURE 4.** (Left) The system used for obtaining of undisturbed soil columns; (right) column leaching studies.

The column experiments were carried out according to the principles described in the literature[17,18,19,20,21,22,23]. Prior to leaching, mine soil column was saturated from the bottom using distilled water at a rate of 8 ml/h; the column remained saturated for 24 h to keep the oxygen content at minimum. The columns were amended with 3,740 kg/ha/year of pig manure, mixed with the soil column from the surface to 10-cm depth. Two doses, 7 and 14%, of the soil weight of 10-cm-thick soil were used. Leachings were carried out weekly using 1,000 ml (50 $l/m^2$) distilled water at a rate of 10 ml/min (Fig. 4b) to simulate critical rainfall events in the area studied (annual average precipitation, 320 mm/year). pH of distilled water was 6.2 at 20ºC. The experiment was carried out for 21 weeks.

Soil columns leachates were collected as a function of time. Leachates were filtered with a Whatman nº 42 paper, and filtered liquid was refrigerated at 5ºC while awaiting chemical analysis. For each week of the experiment, successive aliquots were taken for pH, EC, and Eh analyses. At the end of the each weekly experiment, the amount of leachates were measured and composed for the analyses of pH, EC, Eh, and soluble ions. The amended surface soil material was also analyzed for the same set of analyses.

## RESULTS

The preliminary results from the field plots were: (1) an increase in pH, total nitrogen, organic carbon, and equivalent calcium carbonate contents (Fig. 5); (2) a reduction of DTPA- and water-extractable Zn, Pb, and Cd (Fig. 6 and Fig. 7); and (3) an improvement of plant establishment (Fig. 8), after the addition of organic amendments and lime were observed. Plant density increased with increased doses of pig manure. The addition of pig manure improved the physicochemical soil properties and accelerated the establishment of plants in the sites studied.

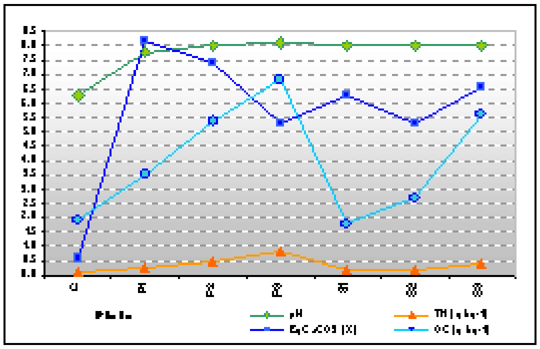

**FIGURE 5.** pH, equivalent calcium carbonate ($EqCaCO_3$), total nitrogen (TN), and organic carbon (OC) values in samples from the plots after 12 months of amendments addition. C: control; pig manure doses P1-P2-P3; sewage sludge doses S1-S2-S3.

At the end of the experiments (21 weeks), pH, EC, and Eh conditions improved in the soil surface in relation to initial soil conditions. Soil pH significantly increased from 2.2 to 3.2 in first application and reached 6.3 in double dose (Fig. 9). Salts diminished significantly from 14.3 to around 1.0 dS/m for both doses, and Eh values increased from 170 to 230 mV.

The pH in the leachate during the experiment suggested that pig manure does not have acid neutralization capability in the short term. In the first leachates, pH was around 1.80 and increased gradually to 2.6 in the single dose and 2.8 for double dose. The opposite behavior was observed for EC, i.e., higher EC was obtained in the initial leachates and decreased progressively from 18.13 to 3.51 and 4.14 dS/m in single and double doses, respectively.

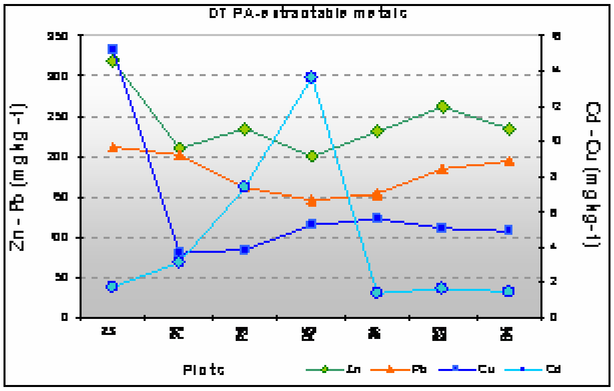

**FIGURE 6.** Concentration of DTPA-extractable metals found in the plots after 12 months of amendments addition. C: control; pig manure doses P1-P2-P3; sewage sludge doses S1-S2-S3.

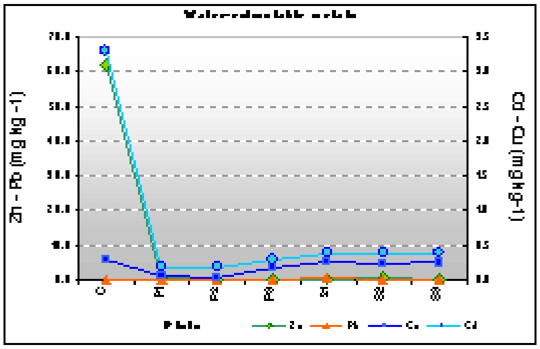

**FIGURE 7.** Concentration of water-extractable metals found in the plots after 12 months of amendments addition. C: control; pig manure doses P1-P2-P3; sewage sludge doses S1-S2-S3.

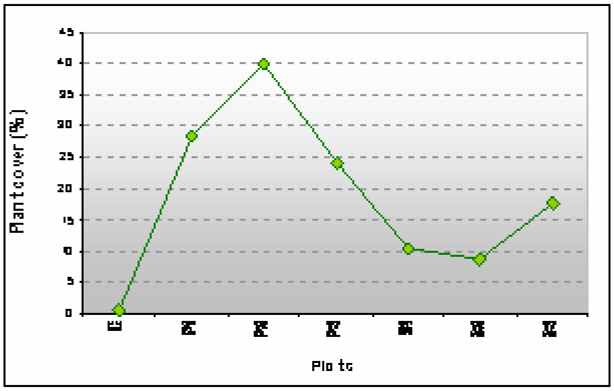

**FIGURE 8.** Percentages of plant cover found in the plots after 12 months of amendments addition. C: control; pig manure doses P1-P2-P3; sewage sludge doses S1-S2-S3.

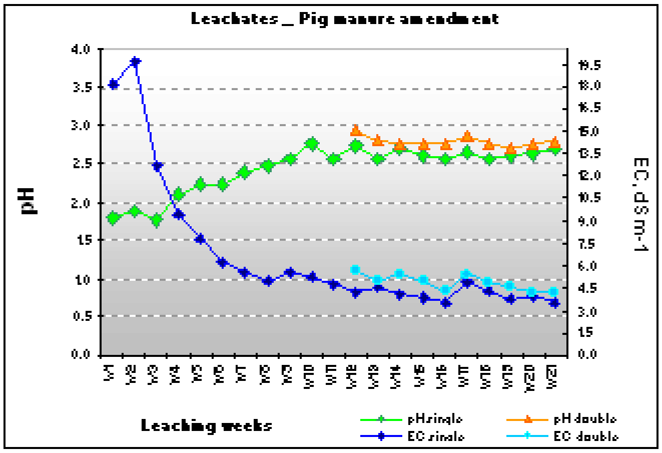

**FIGURE 9**. pH and EC in the leachates from the column leaching studies.

The mobility of metals in the low pH system and high redox potential changed with the addition of pig manure. The total contents of Cd (18 mg/l), Cu (97 mg/l), and Zn (2500 mg/l) in leachates were relatively high at the beginning of the experiment, and decreased steadily over time and reached to 0.1, 3.2, and 22 mg/l, respectively; while Pb increased slightly around 2.0 at week 11 with increasing of pH (Fig. 10). The high metals concentrations may be due to the dissolution of metal-sulfate salts present in the mine soil[24]. The mobility of metals was in the order Zn>Cd>Cu>Pb.

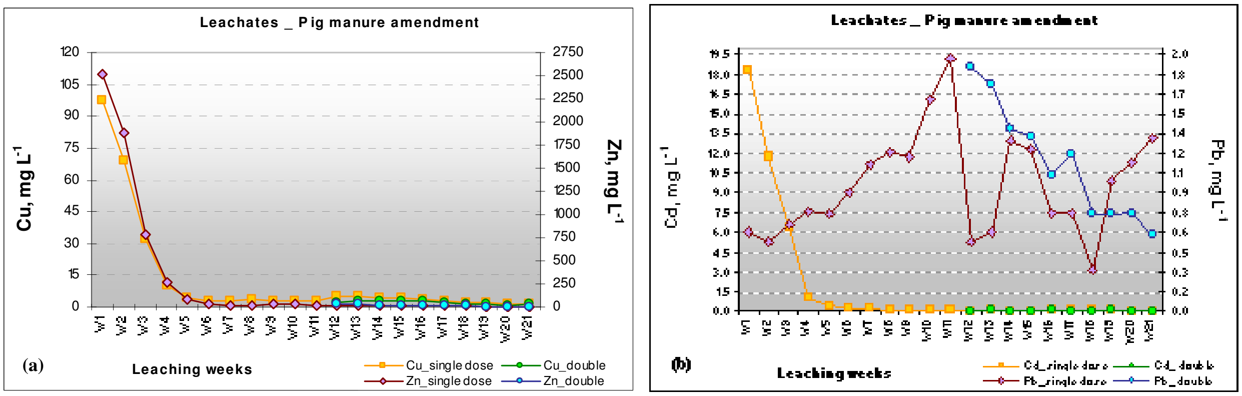

**FIGURE 10.** Evolution of metals concentrations in leachates: Cd-Pb (a) and Cu-Zn (b).

## CONCLUSIONS

The use of the pig manure in mine soils has two advantages: (1) it is a low environmental risk material and (2) it increases the pH and improves the conditions for plant growth. The results obtained in field experiments showed that the addition of pig manure in combination with lime also reduced water- and DTPA-extractable metals, and enhanced the establishment of plants.

The studies showed that pig manure is an environmentally attractive amendment to prevent the formation of acid mine drainage in a long-term remediation program of an abandoned mining site. It seems that application of lime or alkaline materials together with the use of double doses of pig manure is a reasonable alternative for the remediation of mine soils. However, fate of salinity needs to be monitored in medium and long terms.

## ACKNOWLEDGMENTS

To Ministry of Educación y Ciencia (Project PTR1995-0973-OP-02-01 and Project CIT-310200-2005) for the financial support. To Fundación Séneca program of Comunidad Autónoma Región de Murcia.
